# Reversal by Sugammadex

**Published:** 2009-08

**Authors:** Pramila Bajaj

**Affiliations:** Editor, IJA

Reversal agents are often used to ensure the reversal of nondepolarizing neuromuscular blockade (NMB). The most widely used are the acetylcholinesterase inhibitors, neostigmine, and edrophonium. However, these agents are only partially effective against profound NMB, especially in the presence of volatile anaesthetics such as sevoflurane, and may also be associated with adverse effects, such as cholinergic cardiovascular and gastrointestinal events.^1^

Sugammadex is a modified gamma cyclodextrin specifically designed for the reversal of NMB induced by the steroidal neuromuscular blocking agent (NMBA) rocuronium. Sugammadex acts by encapsulating unbound rocuronium molecules and reducing their concentration at the neuromuscular junction^2^ Studies in surgical patients have shown that sugammadex rapidly and safely reverses rocuronium and vecuronium-induced NMB.^3^ Unlike acetylcholinesterase inhibitors, sugammadex is also effective in the reversal of profound NMB.^4^ In previous studies with sugammadex, patients received NMBAs as single or repeat bolus doses. No safety or clinical effect data have been published thus far on sugammadex after continuous infusion of rocuronium, although rocuronium infusion provides stable drug concentrations with a constant degree of paralysis, and its use has become increasingly common^5^. However, continuous infusion of rocuronium has been demonstrated to significantly increase the recovery time after NMB compared with single bolus doses.^6^ In this study by Jellish and coworkers, the median recovery index (time required for the first twitch [T_1_] of the train-of-four [TOF] to recover from 25% to 75% of baseline) after a single bolus dose of rocuronium was 17 min compared with a median recovery index of 24 min after continuous rocuronium infusion. Furthermore, some patients experienced spontaneous recovery times to a TOF ratio of 75% of over 70 min after infusion dosing of rocuronium, suggesting that patients undergoing prolonged infusion are likely to require the administration of a reversal agent.^6^

In clinical practice, rocuronium is administered in combination with volatile agents such as sevoflurane, or with intravenously administered agents such as propofol. The neuromuscular-blocking effect of several NMBAs, including rocuronium, are potentiated under sevoflurane anaesthesia in contrast to propofol-based anesthesia; therefore, the properties of NMBAs under those two anaesthetic regimens have been extensively investigated and compared.^7^,^8^ These studies have shown a delayed recovery induced by accetylcholinesterase inhibitors during sevoflurane anaesthesia as compared to propofol anaesthesia^8^ The effects of sugammadex under maintenance anaesthesia with sevoflurane and propofol have recently been investigated, but only after administration at reappearance of the second twitch of the TOF after a single bolus dose of recuronium (representing a time of partial spontaneous recovery of neuromuscular function).^9^ This situation does differ from continuous infusion, as illustrated by the potentiating effect of volatile anaesthetics, which is clinically most significant during infusion.^7^,^10^ Also, the rapid reversal of sugammadex has been explained by redistribution of rocuronium; during continuous infusion, the ability to redistribute the drug is decreased as a result of distribution compartment saturation.^11^ Thus, the question still remains to be answered whether volatile anaesthetics do influence the clinical effect and safety of sugammadex when used for reversal from neuromuscular blokade induced by continuous infusion of rocuronium.

A single dose of sugammadex 4 mg.kg^−1^ after continuous infusion of rocuronium is equally effective for NMB reversal and well tolerated during maintenance anaesthesia with sevolfurane or propofol. One adverse event (procedural hypotension) is considered to be probably related to sugammadex.
